# Early retirement intentions among Abu Dhabi Police: investigating the role of psychosocial work factors and sickness absenteeism

**DOI:** 10.1186/s12889-023-16129-1

**Published:** 2023-06-23

**Authors:** Faisal Almurbahani Alkaabi, Praveen Kumar Maghelal

**Affiliations:** Faculty of Resilience Rabdan Academy, Abu Dhabi, United Arab Emirates

**Keywords:** Early retirement, Abu Dhabi, Police, Sickness absence, United Arab Emirates

## Abstract

**Background:**

Police departments are encouraged to integrate their health and safety management systems with the operational arrangements to demonstrate commitment to the improvement of working environment in the police is by the collection and analysis of occupational health data such as sickness absence and early retirement intention.

**Methods:**

About 760 responses to the Occupational Health and Safety Survey by the Abu Dhabi Police employees was used to analyse the early retirement intentions considering the work-related factors and sickness absence data.

**Results:**

Logistic regression results of the unadjusted model reported higher odds that lower levels of co-worker support, supervisor support, workplace support related to intentions of early retirement. Also, unfavourable perception of health management increases the odds to early retirement among the employees.

**Conclusion:**

The outcome of this study provides insights into the determinants of early retirement intentions in the less explored region of middle-east, specifically in Abu Dhabi. Thorough analysis of such data will help police organisations to prioritise plans and improve the health and wellbeing of officers, in turn contributing to strengthening the fight against crime and minimizing the number of occupational injuries and premature exit from paid work.

## Background

Police jobs are intrinsically hazardous, where-in studies have shown that policing tasks are light physically but officers are more likely to be involved in stressful situations (mentally demanding) that increase the risk of occupational stress [1]. For instance, some officers, particularly those who are not involved in field work, the majority of working time is spent on sedentary tasks but officers are required to show flexibility and adaptability to respond quickly to sudden changes in the working environment [2]. Occupational stress is associated with sickness absence [3] and early medical retirement [4]. The term ‘ sickness absence’ refers to any absence from work that is attributed to sickness by the employee and accepted as such by the employer [5], while retirement is traditionally defined as “the exit from an organisational position or career path of considerable duration, taken by individuals after middle age, and taken with the intention of reduced psychological commitment to work thereafter” [6].

Also, police officers have a high risk of cardiovascular diseases [7], suicide [8] and occupational injuries [9]. For example, in 2012, the rate of injuries in the UK police force was four times higher than the rate for ‘all occupations’. Similarly, higher rates for police were reported for stress, anxiety and depression [9, 10]. Thus, police departments face difficulties in meeting their legal health and safety obligations as occupational risks are inevitable. Therefore, departments are encouraged to integrate their health and safety management systems with the operational arrangements. A study in UK [2], recommended that one way to demonstrate co-10mitment to the improvement of working environment in the police is by the collection and analysis of occupational health data such as sickness absence and early retirement intention, and is considered a crucial element of organisational reactive health and safety control systems. It is one of the major indicators of organisational continuous commitment for improving the quality of working conditions [11].

Studies that have analyzed the sickness absence and early retirement intentions have grouped its determinants into two groups: work-related and non-work related factors. Work related factors include psychosocial factors, physical characteristics and work arrangements (work shifts) [12], while non-work related factors include age, gender, family factors, socioeconomic status (SES), lifestyle factors, health related factors and other factors such as smoking, drinking and physical activity [13].

In the United Arab Emirates (UAE), the General Directorate of Policing Operations is the largest employer. It is one of the six Directorates divided further into department, branches and units. Abu Dhabi Police is part of the General Directorate of Policing Operations which forms part of the Ministry of Interior of the UAE. It is estimated that Abu Dhabi Police has a workforce of over 35,000 police officers and civilians.

This study focuses on the relation of work-related factors, while controlling for non-work related factors, on early retirement intentions driven by sickness absence. This is one of the first studies investigating this relationship among police officers in the UAE. The outcome of this study provides insights into the determinants of early retirement intentions in the less explored region of middle-east, specifically in Abu Dhabi. Thorough analysis of such data will help police organisations to prioritise plans and improve the health and wellbeing of officers, in turn contributing to strengthening the fight against crime and minimizing the number of occupational injuries and premature exit from paid work.

### Sickness absence and risk of disability retirement

Literature on sickness absence and early retirement intentions report that the total number of sickness absence days increases the risk of disability retirement. Several studies in the last decade have evaluated the relationship between sickness absence and disability retirement using the ‘total number of days of sickness absence’ measure. While some of these used a self-reported measure of sickness absence [14, 15] others utilized register-based data. Two studies used the number of weeks of sickness absence [16] which were translated into days of sickness absence. There was a statistically significant increase in the risk of disability retirement for sickness absence (compared with the reference group in each study) of more than 1d (1 day) [17], 6ds [14], 7ds [18], 15ds [19], 21ds [20], 31ds [15], 90ds [21], 101ds [22] and 281ds [23].

It is worth nothing that most of the studies were conducted in Scandinavian countries which makes it difficult to generalize the findings to other populations since Scandinavian countries have generous social insurance schemes and low wage inequality [24]. Also, majority of the previous studies on the relationship between sickness absence and disability retirement used samples from the general population and not many were from the police force. Therefore, this study fills an important gap in literature by analysing the sickness leave and early retirement intentions in the middle-eastern region.

### Determinants of sickness absence and early retirement intentions

#### Non-work related factors

The non-work related factors that relate to sickness absence include age, gender, family factors, socioeconomic status (SES), lifestyle factors, health related factors and other factors. Literature generally shows that risk of sickness absence increases in females [25], unmarried, divorced, separated and widows/widowers [26], individuals with low socio-economic status (SES) [27]. The research findings on the association between age and sickness absence are mixed [13].

The risk of sickness absence also increases in current and former smokers [28], heavy, frequent and non-alcohol drinkers (the latter also known as ‘abstainers’) [29], problem drinkers [29], obese individuals [30] and physically inactive people [31]. Higher risk of sickness absence is also seen in individuals with the following; poor self-rated health [32], health complaints [13] and low work ability [33].

Similarly, the risk of holding early retirement intentions increases in young employees [34], males [35], more frequent alcohol drinkers and smokers [36], individuals in low SES categories [37] and those reporting poor self-rated health, low work ability [38] or medical symptoms [39].

#### Work related factors

The influence of work factors on sickness absence is evaluated below using the three broad sub classifications of these factors described by Laaksonen et al. [40] and Lahelma et al. [12], namely, psychosocial working conditions (psychosocial exposure), physical working conditions (or physical exposure) and work arrangements.

#### Psychosocial factors

The greater part of the literature investigating the influence of psychosocial work factors on sickness absence is based on the job-demand-control model of Karasek [41]. The influence of high demand on sickness absence are mixed while high job control is shown to reduce sickness absence [25]. Low control [42] and high job demand [43] have been linked to psychological stress and many illnesses that are associated with the increased risk of sickness absence [44]. The combination of the two dimensions (high demand and low control) also known as ‘job strain’ has a stronger effect on health outcomes such as sickness absence than the total effect of the two dimensions considered separately [41, 42].

#### Physical characteristics

The literature review conducted by Allebeck and Mastekaasa [45] on predictors of sickness absence indicated that evidence on the influence of physical work characteristics on sickness absence are ‘limited’, with a poor physical work ergonomic being the strongest of all physical factors as a predictor of sickness absences. Adverse working conditions reduce job satisfaction [33] and cause job stress [46] which in turn, is associated with many illnesses that increase sickness absenteeism [3].

#### Work arrangements

Shift work, particularly evening shift [47], increases sickness absence, because it increases fatigue [15] and conflicts between work and family (Haines et al., 2008) and is linked to many illnesses [48]. The risk of sickness absence may differ between various job types [25]. This relationship is influenced mainly by other factors such as job characteristics [49], socioeconomic status [50], the available support and the representations of genders in different work [51].

Conversely, the available literature provides limited evidence in terms of quantity with respect to the influence of work factors on early retirement intentions. Studies found mixed results regrading influence of job demand on early retirement intention [52, 53] while low job control generally increases early retirement intentions [54]. Low support at work also increases early retirement thoughts [55]. Low job control is associated with many chronic health conditions [56] and may encourage individuals to adopt unhealthy lifestyle behaviours [57] which may in turn, increase the intention to take early retirement.

Finally, as for work arrangement, in general, manual workers have higher risk of holding early retirement intentions than those working in managerial positions [58]. This could be attributed to the physical requirements of the job, the high job demands and low control associated with manual work [49] and the low social support available to these [51].

This study, therefore, builds on current understanding of the role of work-related factors and sickness absence on intentions of retirement among employees of Abu Dhabi police department. Specifically, this study investigates (1) if the psychosocial, physical and employees’ perception of health and safety management system predict early retirement intention in the Abu Dhabi Police after taking into account the influence of age, gender and other covariates, and (2) if employees reporting sickness absence within the last 12 months have an increased risk of early retirement intention compared with their counterparts who reported no sickness absence, with/without taking into account the influence of age, gender and other covariates.

## Method

### Survey administration

An approval to distribute occupational health surveys to Abu Dhabi Police employees was granted by the Secretary General of the Office of His Highness Deputy Prime Minister and Minister of Interior of the UAE. Ethical approval was also obtained from the Health Authority of Abu Dhabi (HAAD) and an official letter was sent from the Human Resource Directorate of Abu Dhabi Police to the Capital Police Directorate to allow the distribution of the surveys to the employees of the latter Directorate. The Directorate in which the study is one of the major Directorates and includes main critical police functions such as investigation, police patrols, police station services and other core functions. These traditional roles induce physical and psychological exposures to police officer that arguably is the same as other international police forces. Some functions of the police services such as ambulance, civil defence, border security at that time were also carried out by the police but were not performed in this Directorates. The Occupational Health and Safety Survey was distributed to 1,317 employees of other departments of the Capital Police Directorate of Abu Dhabi Police between Feb 1st and March 31st 2015. Although the data provided in this study were for the year 2015, the Directorate were not part of any major restructuring or strategic changes which occurred in other Directorates. Therefore, and given the traditional nature of the work, the findings of the study could be generalized to other police Directorates (with similar traditional functions) and international police forces.

The survey was completed by 760 employees (58%); 230 (17%) refused to participate; 259 (20%) did not return the surveys; and 68 (5%) were not surveyed as they were on an authorized long term leave for various reasons (and did not receive the survey). Excluding that last group, the response rate was 61% (760/1,249). Consent to participate was obtained from all the respondents.

### Survey sampling

A liaison officer in the Planning and Research Department of the Directorate was given responsibility for the distribution of surveys in each department of the Directorate. A pilot study was carried out to: evaluate the validity of the Arabic version of the Job Content Questionnaire (JCQ); assess the agreed data distribution procedures; determine sample size required to detect significant difference in the main study and make any necessary changes in relation to the language or format of the questionnaire.

The sample size calculation procedure was conducted in five steps. First, using responses of the pilot study, respondents were classified into low or high occupational exposure categories and the prevalence of the reference group calculated. For job demand, control and support at work, the median of the sample was used to classify participants into low or high exposure categories. In the main study, these three work exposure variables are analysed using three levels and it was anticipated that the power would be roughly the same since we were comparing more extreme groups and the OR may be expected to be higher than we are assuming when comparing just two groups. For physical work exposures, participants were considered to have high exposure if they were exposed to such hazards for half or more of their working time. Secondly, odds ratios for the relationship between each work-related variable and sickness absence were obtained from the literature and used as reference values that were expected to be found between different exposure groups in the main survey.

Thirdly, the estimated prevalence of outcome (having sickness absence of ≥ 4d) in the reference group for each work exposure was calculated, such that the prevalence of the outcome, with the given odds ratio, in the whole sample is the same as that observed in the whole pilot study (23%). Fourthly, the power was set at 80% and level of significance set at the default value of 5% and the sample size calculated.

Finally, after obtaining the sample size for each work related variable, an inflation factor of 39% was added to account for potential non respondents.

Table 1 shows the sample size required to detect statistically significant differences, with 80% power, between various exposure groups in the main survey using the pilot study results. The table also shows the numbers used in the calculation of the sample size for each work exposure variable for each of the five steps above. In general, as the main survey is distributed to the remaining employees of the Directorate (1,425–108 = 1,317), the sample size required to detect differences between groups with various work exposures at the given power of 80% is achieved for all the variables, except for exposure to vibration. Therefore, except for exposure to vibration, the main survey has over 80% power to detect differences, at the 5% level of significance, between groups with various work exposures.

**Table 1:** Sample size required to detect statistical difference, at the 5% level of significance, with 80% power in the main survey, using estimates from the pilot study

Work related variable	Prevalence of SA outcome in reference group^a^		*p1* (%)^b^	Odds ratio^c^	Sample size required	Adjusted sample size ^d^
*Job demand*		0.18	50	1.70	*694*	***965***
*Job control*		0.18	50	1.80	*562*	***781***
*Work place support*		0.18	50	1.68	*726*	***1009***

^a^This is chosen so that the overall prevalence of sickness absence in the whole study is 23%, the observed prevalence of having sickness absence of ≥ 4d in the pilot study

^b^This is the prevalence of the control or reference group (not exposed). It was assumed that we have equal size of groups for job control, job demand and workplace support as these variables are split by the median into low and high categories

^c^Odds ratio value from the literature

^d^Sample size required after allowing for non-response

### Study variables

#### Outcomes/dependent variable: Early retirement intention

As with Von Bonsdorff et al. [34] and Neacsiu [59], early retirement intentions were assessed by asking participants if they had ever thought of retiring before reaching retirement age with the following responses, ‘Never’, ‘Yes, often’, ‘Yes, sometimes’ and ‘I have already applied’. During statistical analysis, participants were categorized into either holding (‘Yes, often’, ‘Yes, sometimes’ and ‘Already applied’) or not holding intentions to retire early.

In the UAE, the retirement age is 60 for males and 55 for females, however, government employees are entitled to apply for early exit from paid work after completing 20 years of service. Therefore, the early retirement intention question was reformulated to ask whether participants intend to take early retirement after completing the years of service that make them eligible for early exit and before reaching the full legal retirement age. Similar to earlier studies, participants who reported an intention to retire early were asked to specify the primary reason for such intention [60].

### Sickness absence

Participants were asked about the number of days of sickness absence in the last 12 months. Response options were none, 1-3d, 4-7d, 8-28d, 29-90d and ≥ 91d. This self-reported sickness absence measure has been used in many previous studies [61, 62]. For this study, the last four responses in the sickness absence outcome were combined into one category (≥ 4d), grouping the respondents into three categories: 0 days; 1–3 days and ≥ 4 days.

### Psychosocial work factors

Many studies have used the Karasek’s JCQ in different languages including South Korean [63], Brazilian [64], Persian [65] and other languages. Thus, it was decided to use this measure to evaluate the influence of psychosocial work factors on sickness absence and early retirement intention on our police sample. However, as the JCQ had not yet been translated into the Arabic language, this study aimed to develop an Arabic version of this questionnaire (the Arabic JCQ) to survey the Abu Dhabi Police, one of the largest organization in the Middle East.

These include 22 psychosocial items of the 49 items of the JCQ of Karasek’s [66] model. Nine of these items were for job control, five job demand items, and eight workplace support items. These measures have been used previously to examine the risks of job control, demand and support on sickness absence [61, 62] and early retirement intentions [59]. The questionnaire was translated into Arabic for ease of response as majority of employees in police are Arabic natives.

The distribution of responses for the job control, demand and support variables were used to classify participants to low, medium and high categories. Participants were then arranged into four psychosocial work-status categories, namely, active (high control/high demand), passive (low control/low demand), relaxed (high control/low demand) and job strain (low control/high demand).

### Physical work exposures

The physical work items in the survey included questions regarding intensity of nine physical exposures with questions based on the European Working Conditions Survey [67]. Nine physical work factors; vibration, noise, working in painful positions, lifting people, carrying heavy objects, working while standing, work requiring repetitive hand-arm movements, working on screens and dealing with angry clients were inquired. Participants were categorized into being either exposed less than half of the time (low) or half of the time or more (high). A combined physical exposure variable was created by adding the low (1) and high (2) responses from the nine physical exposure variable and then using the distribution of data to create low, moderate and high physical exposure categories.

### Perceptions of organisational health and safety management systems

Using the UK Health and Safety Executive’s guide for successful health and safety management system [11], participants were asked seven questions regarding their perception of the health and safety management systems implemented by the Abu Dhabi Police. An item reflecting overall perception of health and safety management system was also created (from the aforementioned items, HSMST). Participants were categorized into having favourable (strongly agree and agree) and unfavourable (strongly disagree and disagree) perceptions.

### Other covariates

Other covariates included demographic variables (Age, gender, etc.), health indicators (self-rated health, exercise, etc.), lifestyle (smoking, work ability, etc.), social life factors (number of children, work-life balance, etc.) and work setting (work shift, rank, etc.).

### Data analysis

Descriptive analyses included the overall distribution of responses for each subcategory of work in relation to intent to retire early. The risk of sickness absence and early retirement intentions were then evaluated in relation to psychosocial work factors, employees’ overall perception of health and safety management system (HSMST) and the combined physical exposure variable (as this study primarily focuses on these factors) using two models for the adjustment of covariates. This first stage of the analysis compared the unadjusted odds ratio estimates with those from Model I, which adjusted for age and gender, and Model II which additionally adjusted for other demographic factors that are associated with the outcome. These analyses were carried out using the restricted sample of responders with no missing values for any of the covariates in Model II.

A second stage of the analysis incorporated three additional models. Each included all the covariates in the preceding model. As with Model II, covariates were added only if they were also associated with the outcome. Model III additionally included social life, health and lifestyle factors, while work setting factors were also added in Model IV. The fully adjusted model added the psychosocial and physical factors as well as employees’ perception of health and safety management system. The analyses in this stage were conducted using the restricted sample of the fully adjusted model.

Finally, the influence of various self-reported durations of sickness absence on the risk of holding early retirement intention compared with not intending to retire early was evaluated using logistic regression. This was carried out using the full sample and the restricted samples of model II and the fully adjusted model.

## Results

This section provides descriptive statistics regarding distribution of work factors and their (unadjusted) association with early retirement intentions. This study evaluates only the following work factors; psychosocial work factors, overall perception of health and safety management system and combined physical exposure.

### Work factors and the risk of early retirement intention

Thirty-eight percent of respondents reported early retirement intentions (Table 2). Of the 253 police employees who intended to retire early, 239 provided the main reason for their retirement. About 53% (127/239), reasoned that it was to ‘enjoy life and spend more time with partner’ or because they were ‘fed up with job and want a change (possibly another job)’. Less than 10% held this intention due to their ‘own ill-health’ or ‘ill health of a relative/friend’.

**Table 2:** Overall distribution of work factors and early retirement intention

Early retirement intention (ERI)					
**Work factor**	**n (%)**	**n with ERI data**	**No n (%)**^**b**^	**Yes n (%)**	**Odds Ratio**^**a**^** (95% CI)**
***Early Retirement Intention***		663	410 (62)	253 (38)	
***Job control***					
High	202 (30)	173	121 (70)	52 (30)	1
Medium	230 (34)	211	137 (65)	74 (35)	1.25 (0.81–1.93)
Low	252 (37)	229	131 (57)	98 (43)	**1.74 (1.14–2.16)**
* Total*	*684 (100)*	*613*	*389 (63)*	*224 (37)*	
***Job demand***					
Low	221 (31)	190	116 (62)	74 (38)	1
Medium	265 (37)	224	149 (61)	95 (39)	0.95 (0.64–1.40)
High	238 (33)	209	139 (67)	70 (33)	0.75 (0.50–1.13)
* Total*	*724 (100)*	*643*	*404 (62)*	*239 (37)*	
***Co-workers support***					
High	250 (35)	213	153 (72)	60 (28)	1
Medium	317 (44)	293	182 (62)	111 (38)	**1.55 (1.06–2.27)**
Low	153 (21)	136	64 (47)	36 (53)	**2.95 (1.88–4.63)**
* Total*	*720 (100)*	*642*	*399 (62)*	*243 (38)*	
***Supervisors support***					
High	281 (38)	244	171 (70)	73 (23)	1
Medium	284 (39)	262	163 (62)	99 (38)	1.42 (0.98–2.06)
Low	168 (23)	144	68 (47)	76 (53)	**2.61 (1.70–4.01)**
* Total*	*733 (100)*	*650*	*402 (62)*	*248 (38)*	
***Workplace support***					
High	265 (37)	230	168 (73)	62 (27)	1
Medium	295 (41)	273	162 (59)	111 (41)	**1.85 (1.27–2.71)**
Low	152 (21)	131	62 (47)	69 (53)	**3.01 (1.92–4.72)**
* Total*	*712 (100)*	*634*	*392 (62)*	*242 (38)*	
***Psychological work classification***					
Relaxed	135 (20)	122	85 (70)	37 (30)	1
Passive	302 (45)	273	161 (59)	112 (41)	**1.59 (1.01–2.51)**
Active	117 (18)	96	71 (74)	25 (26)	0.80 (0.44–1.46)
Strain	114 (17)	107	66 (62)	41 (38)	1.42 (0.82–2.46)
* Total*	*668 (100)*	*598*	*383 (64)*	*215 (36)*	
***Overall perception of HSE management system (HSMST)***					
Favourable	561 (78)	498	328 (66)	170 (34)	1
Unfavourable	175 (22)	144	70 (49)	74 (51)	**2.03 (1.40–2.96)**
* Total*	*718 (100)*	*642*	*398 (62)*	*244 (38)*	
***Combined physical exposure***					
Low	233 (37)	217	153 (71)	64 (29)	1
Moderate	188 (30)	166	105 (63)	61 (37)	1.38 (0.90–2.13)
High	215 (34)	192	98 (51)	94 (49)	**2.29 (1.52–3.44)**
* Total*	*636 (100)*	*575*	*356 (62)*	*219 (38)*	

^a^Unadjusted odds ratio of reporting intention for retiring early compared with those without such intention

^b^Row percentages

In the unadjusted model, a statistically significant increase in the odds of early retirement intention was seen in police employees in the low versus high categories of all psychosocial work factors, apart from job demand which had a statistically non-significant protective effect. The increase in the odds of early retirement intention ranged from OR = 1.74 (95%CI = 1.14–2.16) in the low job control category to OR = 3.01 (95%CI = 1.92–4.72) in those reporting low workplace support (Table 2).

Statistically significant increases in the odds of early retirement intention by 55% and 85% were also seen in the medium versus high category of co-workers support and workplace support respectively. Participants with ‘passive’ psychological work status had a rise in the odds of early retirement intention compared with those in the ‘relaxed’ category (OR = 1.59, 95%CI = 1.01–2.51).

Significant increase in the odds of early retirement intention was seen in police employees with high (versus low) combined physical exposures (OR = 2.29, 95%CI = 1.52–3.44). Statistically significant increased odds of early retirement intentions were seen in participants who were exposed for at least half of their working time to any of six physical work factors: vibration, noise, working in painful positions, lifting people, working on screens and dealing with angry clients.

A twofold increase in the odds of early retirement intentions was seen in participants reporting unfavourable overall perception of the health and safety management system (HSMST) (Table 2). For seven of the health and safety dimensions (excluding HSMS5), individuals with unfavourable perceptions also had significantly increased odds of early retirement intention.

### Adjustment models

Table 3 shows the odds ratio of the association between work factors and the risk of early retirement intention in three models. Education, marital status and years of service were also associated with early retirement intention and were included in model II.

**Table 3:** Work factors and the odds ratio of early retirement intention in three statistical models^a^

Work factor	Unadjusted	Model I	Model II
***Job control (n* = *523)***			
High	*Reference*		
Medium	1.33 (0.84–2.12)	1.45 (0.90–2.32)	1.49 (0.92–2.41)
Low	1.45 (0.92–2.28)	1.50 (0.94–2.39)	1.55 (0.97–2.49)
***Job demand (n* = *536)***			
Low	*Reference*		
Medium	1.09 (0.69–1.71)	1.17 (0.74–1.87)	1.22 (0.76–1.96)
High	0.99 (0.62–1.58)	1.03 (0.64–1.67)	0.96 (0.58–1.56)
***Psychological work classification (n* = *511)***			
Relaxed	*Reference*		
Passive	1.26 (0.77–2.07)	1.37 (0.83–2.27)	1.33 (0.80–2.21)
Active	0.71 (0.37–1.34)	0.72 (0.37–1.38)	0.61 (0.31–1.19)
Strain	1.48 (0.83–2.63)	1.57 (0.87–2.82)	1.44 (0.79–2.63)
***Co-workers’ support (n* = *538)***			
High	*Reference*		
Medium	1.43 (0.95–2.17)	1.39 (0.91–2.13)	1.45 (0.94–2.23)
Low	**2.32 (1.40–3.80)**	**2.29 (1.37–3.82)**	**2.53 (1.48–4.30)**
***Supervisors’ support (n* = *543)***			
High	*Reference*		
Medium	1.49 (0.99–2.23)	1.45 (0.96–2.20)	1.56 (0.99–2.39)
Low	**2.14 (1.32–3.48)**	**2.17 (1.33–3.57)**	**2.35 (1.41–3.92)**
***Workplace support (n* = *533)***			
High	*Reference*		
Medium	**1.91 (1.27–2.28)**	**1.83 (1.20–2.78)**	**1.88 (1.22–2.89)**
Low	**2.25 (1.35–3.76)**	**2.26 (1.34–3.80)**	**2.47 (1.44–4.22)**
***Overall perception of HSE management (n* = *536) (HSMST)***			
Favourable	*Reference*		
Unfavourable	**1.78 (1.16–2.71)**	**1.66 (1.08–2.55)**	**1.67 (1.08–2.59)**
***Combined physical exposure (n* = *483)***			
Low	*Reference*		
Moderate	1.39 (0.87–2.20)	1.38 (0.86–2.22)	1.39 (0.85–2.25)
High	**1.66 (1.05–2.61)**	**1.90 (1.18–3.03)**	**1.92 (1.18–3.11)**

Model I: Adjusted for age and gender

Model II: Previous model + education, marital status and years of service

^a^Using the restricted sample of model II with no missing covariate data

There was a statistically significant increase in odds of early retirement intention in the three models in the low and medium (versus high) categories of the three workplace support variables and those with unfavourable (versus favourable) perception of health and safety management system. The adjustment did not make large difference in the associations. The odds of early retirement intention in police employees with high (versus low) combined physical exposures increased significantly by 66%, 90% and 92% in the unadjusted, model I and model II respectively.

Participants with low and medium (versus high) job control had a statistically non-significant increase in odds of early retirement intention in all three models. Similar non-significant associations in the three models were seen in those with medium and high (versus low) job demand. Compared with those in the ‘relaxed’ psychological work status (reference group), police employees in the ‘passive’ and ‘strain’ categories had a statistically non-significant raise in the odds of early retirement intention in all three models with an increase of 33% and 44% in model II respectively while those in the ‘active’ job category had a non-significant protective effect in the three models.

Using the restricted sample of the fully adjusted model (Table 4), the influence of work factors on the odds of early retirement intention was similar to that seen in the unadjusted model (Tables 2). A comparison between the influence of work factors on the odds of early retirement intention in the unadjusted model and model I in the full sample and the restricted sample of the fully adjusted model is show two main differences. First, participants with low (versus high) job control, medium (versus high) co-workers’ support, ‘passive’ compared with ‘relaxed’ psychological work status and high (versus low) combined physical exposures had a significant increase in the odds of early retirement intention in the full sample (Table 2) which remained increased but with no statistical significance in the sample of the fully adjusted model (Table 4). Secondly, police employees with high (versus low) job demand had a non-significant protective effect of early retirement intention in the unadjusted model and model I in the full police sample.

**Table 4:** Work factors the odds ratio of early retirement intentions (ERI) in six statistical models^a^

Work factor	Unadjusted	Model I	Model II	Model III	Model IV	Fully adjusted
***Job control (n* = *319)***						
High	*Reference*					
Medium	1.03 (0.56–1.89)	1.00 (0.54–1.85)	1.03 (0.55–1.93)	1.11 (0.51–2.41)	1.29 (0.58–2.88)	0.92 (0.37–2.29)
Low	1.54 (0.88–2.72)	1.45 (0.82–2.59)	1.50 (0.84–2.70)	1.22 (0.59–2.54)	1.35 (0.62–2.93)	1.42 (0.59–3.42)
***Job demand (n* = *328)***						
Low	*Reference*					
Medium	1.31 (0.71–2.31)	1.35 (0.73–2.52)	1.43 (0.75–2.72)	1.79 (0.83–3.87)	1.81 (0.81–4.00)	1.70 (0.70–4.13)
High	1.06 (0.58–1.95)	1.09 (0.58–2.06)	0.99 (0.52–1.88)	0.94 (0.43–2.04)	0.92 (0.41–2.04)	0.91 (0.38–2.21)
***Psychological work classification (n* = *315)***						
Relaxed	*Reference*					
Passive	1.29 (0.69–2.41)	1.31 (0.69–2.49)	1.38 (0.72–2.61)	1.23 (0.56–2.71)	1.57 (0.68–3.63)	1.37 (0.54–3.50)
Active	0.74 (0.34–1.62)	0.77 (0.34–1.71)	0.69 (0.30–1.58)	0.49 (0.18–1.35)	0.55 (0.20–1.53)	0.48 (0.15–1.52)
Strain	1.43 (0.70–2.91)	1.44 (0.69–3.01)	1.29 (0.61–2.73)	1.04 (0.42–2.57)	1.16 (0.46–2.91)	1.23 (0.43–3.46)
***Co-workers support (n* = *315)***						
High	*Reference*					
Medium	1.65 (0.96–2.86)	1.44 (0.82–2.53)	1.46 (0.82–2.60)	1.95 (0.95–4.02)	2.21 (0.98–4.68)	2.17 (0.88–5.32)
Low	**2.76 (1.38–5.17)**	**2.32 (1.18–4.56)**	**2.50 (1.23–5.07)**	1.60 (0.66–3.87)	1.72 (0.70–4.26)	1.86 (0.62–5.53)
***Supervisors support (n* = *315)***						
High	*Reference*					
Medium	1.69 (0.99–2.90)	1.57 (0.90–2.72)	1.65 (0.93–2.92)	1.65 (0.80–3.39)	1.67 (0.79–3.53)	1.19 (0.48–2.93)
Low	**2.14 (1.14 -4.01)**	**2.10 (1.11–3.97)**	**2.16 (1.11–4.18)**	0.97 (0.41–2.27)	0.92 (0.38–2.22)	0.55 (0.19–1.60)
***Workplace support (n* = *315)***						
High	*Reference*					
Medium	**2.20 (1.28–3.80)**	**1.94 (1.11–3.39)**	**1.97 (1.11–3.49)**	**2.21 (1.06–4.62)**	**2.19 (1.02–4.69)**	**2.42 (1.01–5.87)**
Low	**3.52 (1.30–4.88)**	**2.35 (1.20–4.60)**	**2.49 (1.25–4.98)**	1.17 (0.47–2.86)	1.15 (0.46–2.88)	1.00 (0.35–2.85)
***Overall perception of HSE management (n* = *313)***						
Favourable	*Reference*					
Unfavourable	**2.05 (1.20–3.49)**	**1.84 (1.07–3.19)**	**1.82 (1.04–3.17)**	1.30 (0.65–2.59)	1.19 (0.57–2.46)	1.43 (0.61–3.34)
***Combined physical exposure (n* = *315)***						
Low	*Reference*					
Moderate	1.62 (0.91–2.85)	1.58 (0.88–2.85)	1.70 (0.94–3.09)	1.60 (0.78–3.27)	1.79 (0.85–3.78)	1.97 (0.88–4.40)
High	1.48 (0.84–2.60)	1.68 (0.94–3.00)	1.78 (0.97–3.24)	1.50 (0.70–3.19)	1.53 (0.70–3.33)	1.81 (0.76–4.28)

Model I: Adjusted for age and gender

Model II: Previous model + education, marital status and years of service

Model III: Previous model + number of children, work life imbalance, private life support frequency of meeting friends, tragic events, self-rated health, work ability, presenteeism, BMI category, smoking status and exercise

Model IV: Previous model + work hours and transportation time to work

Fully adjusted: previous model + control, demand, support, vibration, noise, working in painful position, lifting, working on screens, dealing with angry clients and all factors relating to perceptions of occupational health and safety management systems

^a^Using the restricted sample of the fully adjusted model with no missing covariate data

The odds of early retirement intention remained statistically significant in all models only in police employees with medium (versus high) workplace support. A marked but non-significant increase in the odds of early retirement intention was seen in model III for participants with medium (versus high) co-workers’ support (49%) and medium (versus low) job demand (36%). In participants in the low (versus high) categories of the three support variables and those with unfavourable (versus favourable) overall perception of the health and safety management system (HSMST), the statistically significant increase in the odds of early retirement intention decreased between the unadjusted model to model II and became raised non-significantly from model III (Table 4).

### Summary of findings

This study evaluated the influence of psychosocial work factors, physical work factors and employees’ perception of organisational health and safety management system on the risk of sickness absence and early retirement intentions. The study also evaluated the relationship between self-reported sickness absence and early retirement intention.

The risk (odds) of having sickness absence of four days or more (≥ 4d) compared with 0 to 3 days (0-3d) and the risk of holding early retirement intention compared with not intending to retire early were estimated using logistic regression. Psychosocial work factors and perceptions of health and safety management systems were not significant predictors of sickness absence in this police sample. Surprisingly, officers who reported high combined physical exposures (compared with those reporting low) had a significantly protective effect from sickness absence after adjusting for all covariates.

Low job control resulted in a significant increase in the risk of early retirement intentions in the unadjusted model of the full sample. However, the relationship became raised non-significantly when the restricted sample of model II or the fully adjusted model sample were used. Job demand, strain and workplace support were not significant predictors of early retirement intentions.

Officers reporting unfavorable overall perception of health and safety management system had a significant increase in the risk of early retirement intentions. This relationship became non-significant after adjusting for social, health and lifestyle factors. Officers with high combined physical exposures also had a significant increase in the risk of early retirement intention and the relationship also became non-significant after adding various adjustments and using the restricted sample of the fully adjusted model.

Finally, using the full sample of this study, there was a significant increase in the risk of early retirement intention in officers with ≥ 4d of sickness absence (compared with those reporting no sickness absence days). The association remained significant when using the restricted sample of model II in the unadjusted model as well as after adjusting for age, gender and other demographic covariates. However, when the restricted sample of the fully adjusted model was used, the risk of early retirement intention increased significantly in officers reporting ≥ 4d of sickness absence only in the unadjusted model.

### The relationship between sickness absence and early retirement intentions

Participants who reported ≥ 4d of sickness absence in the past 12 months were more likely to intend to retire early (Table 5). A statistically significant increase in the odds of early retirement were seen only in participants reporting ≥ 4d of sickness absence (OR = 1.79, 95%CI = 1.22–2.63) compared with the reference group (0d). Individuals reporting 1-3d had a non-significant increase (OR = 1.37, 95%CI = 0.92–2.02).

**Table 5:** Sickness absence durations in the last 12 months and early retirement intention (full sample)

Early retirement intention (ERI)					
**Factor**	**n (%)**	**n with ERI data**	**No n (%)**^b^	**Yes n (%)**	**Odds Ratio**^a^** (95% CI)**
**Full sample**		663	410 (62)	253 (38)	
***Sickness absence***					
0d	340 (51)	327	220 (67)	107 (33)	1
1-3d	161 (24)	160	96 (60)	64 (40)	1.37 (0.92–2.02)
≥ 4d^c^	169 (25)	165	88 (53)	77 (47)	**1.79 (1.22–2.63)**
* Total*	*670 (100)*	*652*	*404 (62)*	*248 (38)*	

^a^Unadjusted odds ratio of reporting intention for retiring early compared with those without such intention

^b^Row Percentages

^c^Categories 4-7d and ≥ 8d were combined due to small sample of the latter category (10%)

The unadjusted odds ratios using the restricted sample of model II (Table 6) and the sample fully adjusted model (Table 7) for police employees with ≥ 4d of sickness absence were similar to that seen in the full sample (Table 5). The results for models I and II in Tables 6 and 7 were also similar, although not statistically significant when using the restricted sample of the fully adjusted model.

**Table 6:** Sickness absence durations in the last 12 months and the odds ratio of early retirement in three statistical models^a^

Factor	Unadjusted	Model I	Model II
***Sickness absence (n* = *547)***			
0d	*Reference*		
1-3d	1.18 (0.76–1.84)	1.39 (0.88–2.18)	1.41 (0.89–2.25)
≥ 4d	**1.82 (1.19–2.79)**	**1.90 (1.22–2.95)**	**1.89 (1.20–2.97)**

Model I: Adjusted for age and gender

Model II: Model I + education, marital status and years of service

^a^Using the restricted sample of model II with no missing covariate data

**Table 7:** Sickness absence durations in the last 12 months and the odds ratio of early retirement in six statistical models^a^

Work factor	Unadjusted	Model I	Model II	Model III	Model IV	Fully adjusted
***Sickness absence (n* = *316)***						
0d	*Reference*					
1-3d	0.98 (0.55–1.74)	1.06 (0.59–1.92)	1.07 (0.58–1.95)	0.96 (0.47–2.00)	0.92 (0.43–1.96)	0.89 (0.38–3.10)
≥ 4d	**1.81 (1.04–3.17)**	1.76 (0.99–3.12)	1.70 (0.94–3.04)	1.61 (0.80–3.22)	1.65 (0.80–3.39)	1.74 (0.76–3.99)

Model I: Adjusted for age and gender

Model II: Previous model + education, marital status and years of service

Model III: Previous model + number of children, work life imbalance, private life support, frequency of meeting friends, tragic events, self-rated health, work ability, presenteeism, BMI category, smoking status and exercise

Model IV: Previous model + work hours and transportation time to work

Fully adjusted: previous model + control, demand, support, vibration, noise, working in painful position, lifting, working on screens, dealing with angry clients and all factors relating to perceptions of occupational health and safety management systems

^a^Using the restricted sample of the fully adjusted model with no missing covariate data

## Discussion and conclusion

The association between self-reported sickness absence and the risk of early retirement intention was evaluated. The number of days of sickness absence; the results have shown that officers reporting ≥ 4d of sickness absence (versus 0d) within the last 12 months had a significant increase in the risk of early retirement intention but not officers with 1-3d. There is only one previous study by Heponiemi et al. [38] which examined the association between sickness absence and the risk of early retirement intentions. The study used a binary sickness absence measure (Yes/No) and also found that those reporting sickness absence had a significant increase in the risk of reporting early retirement intentions. Therefore, it can be argued that the current study partially agrees with the study because officers with 1-3d of sickness absence did not a significant increase in the risk of holding early retirement intention.

In the full sample of this study, there was a significant increase in the risk of early retirement intention in officers with ≥ 4d of sickness absence compared with those reporting no sickness absence days in the last 12 months. The association remained significant when using the restricted sample of model II and after adjusting for age, gender and other demographic covariates. This is in line with the findings of the only study that evaluated this relationship [38]. However, the latter study used a self-reported binary measure of sickness absence (Yes/No) while the current study included various sickness absence durations.

Sickness absence is negatively associated with job satisfaction [68] which was also associated with early retirement intentions [69]. Thus, police officers with various sickness absence durations could have been dissatisfied about their work which in turn, may explain the increase in risk of holding early retirement intentions.

Previous studies have shown that job satisfaction is considered as a strong predictor of early retirement intentions [55]. The lack of association between short term sickness absence (1–3 days) and early retirement intentions could be explained by the high satisfaction of officers with other job aspects. For example, Heponiemi et al. [38] showed that organizational injustice strengthens the relationship between sickness absence and the risk of early retirement intentions. Thus, officers reporting 1–3 days of sickness absence may perceive high organizational justice which weakens the association between sickness absence and early retirement intentions.

Evaluating other closely related factors such as organisational injustice and job control may also provide more insights into the relationship between sickness absence and early retirement intention. For example, Heponiemi et al. [38] found that organisational injustice strengthens the association between sickness absence and early retirement intentions. Unfortunately, this variable was not included in the survey. It can also be argued that officers may take ≥ 4d of sickness absence as a coping strategy to deal with their stressful working environment or dissatisfying jobs. Job satisfaction is a significant predictor of early retirement intentions [55].

Using the restricted sample of the fully adjusted model, the risk of early retirement intention increased significantly in officers reporting ≥ 4d of sickness absence only in the unadjusted model. It increased but not significantly after adjusting for age and gender. This could be attributed to the strong influence of demographic variables, age in particular, on early retirement intention. This mainly include age and years of service which are also associated with sickness absence (analysis not shown). Older officers in this sample were more likely to take long rather than short durations of sickness absence. For example, 14% and 8% of those between 40 and 49 years of age took ≥ 8d and 1-3d of sickness absence respectively compared with 8% and 24% respectively for officers between 30 and 39.

In the current research, when using the restricted sample of the fully adjusted model, the relationship between sickness absence and the risk of early retirement intention became non-significant when adjusted for age and gender. As sickness absence predicted early retirement intention only in the unadjusted model, the findings of this research only partially agree that employees reporting sickness absence in last 12 months have increased risk of early retirement.

As individuals age, their health declines and they tend to take sick leave more frequently in general. In addition, with older age, work ability reduces and employees might take sickness absence more often to recover from the negative consequences of work exposures. Increase in age is hence a potential confounder for the relationship between sickness absence and early retirement intention. Furthermore, it can be argued that with older age, employees are likely to have strong early retirement intentions as they get fed up with their working career and their priorities change. In general, this research adds that sickness absence of ≥ 4d predicts early retirement intention while sickness absence of 1-3d does not. Long term sickness absence is typically associated with long standing or chronic illnesses which may reduce employees working ability. Officers with long term sickness absence may have an increased risk of early retirement intention because they believe that their working ability has declined and that work might cause further health problems [70].

### Implications for practice

The study provides many suggestions for the Abu Dhabi Police and for law enforcement organisations in general. Police organisations must demonstrate continuing commitment to the improvement of working conditions of police employees by allowing researchers to evaluate the influence of work factors on various health and non-health outcome. This will ensure that work exposures specific to the policing environment are identified thoroughly, which, in turn, will allow for the effective planning and implementation of appropriate health and safety control measures.

Furthermore, police employees may feel reluctant to give honest responses in surveys to avoid being identified and potentially discriminated against or even re-allocated. Therefore, future surveys could be distributed by the police union rather than the management, to minimise bias in responses. This research has also shown that other factors—such as job satisfaction, job involvement and social factors (such as private life support and frequency of meeting friends) may explain differences in results between the findings of this research and the literature in relation to work predictors of sickness absence and early retirement intentions. Therefore, police organisations are advised to continuously improve employees’ satisfaction and social networking (inside and outside the organisation) to minimise the influence of work factors on this intrinsically hazardous occupation.

This research also showed that number of days of sickness absence is a good predictor of early retirement intentions. Therefore, either of these measures could be used as early indicators when setting plans to minimise disability retirement cases. It is also advisable that the Abu Dhabi Police Medical Committee broadens its duties to include a formal return to work assessment after certain sickness absence periods. For example, employees with a sickness absence of ≥ 4 weeks could be interviewed prior to returning to work to check if they are fit physically and psychologically and to discuss openly the need for further sick leave or other actions or interventions to reduce the likelihood of future sickness absences.

Moreover, police organisations must ensure that appropriate channels are designed and implemented for police employees to seek help when faced with work problems. These channels should maintain anonymity, should not result in re-allocation to undesired posts nor have negative financial consequences and should conform to employees’ expectations and needs. For example, the Abu Dhabi Police could establish independently run employee assistance programmes that ensure anonymity and provide care and well-being advice and interventions delivered by staff from various backgrounds such as occupational health psychologists, human resource experts and other career experts.

Finally, in the Abu Dhabi Police, despite evaluating the association between sickness absence and early retirement intentions, the management were not in favour of this approach to avoid giving an indication of the general demographic characteristics of the organisation. Thus, the management of the Abu Dhabi Police is advised to establish a research committee that evaluates any request to conduct research using employees’ data from researchers working in the police or other organisations and also provide research opportunities and data for academics and policy-makers. Further studies could be conducted to reduce the time-bound challenge associated with cross-sectional studies by potentially developing longitudinal studies. The Abu Dhabi Police should also use research findings during the development of occupational policies and ensure appropriate implementation of policies into practice.

There are four main limitations of this study. This study used self-reported measures which are subject to recall bias as in other similar studies [71]. The cross-sectional design of the study makes it difficult to establish causal inference: if the exposure to work factors led to the outcome or the relationship exist in the opposite direction [72]. Missing data was another limitation of this study. It resulted in reducing the sample size to almost half when adjusting for various covariate groups. Finally, females were under-represented in this sample of police officers which made it difficult to carry out stratified analyses of results.

Barring these limitations, this study bridges the gap in the literature regarding work predictors of sickness absence and early retirement intentions in the police force. Another strength of this study is that it is one of the first to evaluate the influence of employees’ perception of organisational health and safety management system on the risk of sickness absence and early retirement intention [73]. The study has a high response rate and is also one of very few to investigate the use of sickness absence data in the prediction of early retirement intention. As this study was carried out in one of the largest organisations in the UAE, the findings of the study could be compared with future occupational health research in the Middle East.

## Data Availability

The datasets generated and/or analyzed during the current study are not publicly available due to privacy and the sensitivity of the data (as per UAE regulations) and are are available from the corresponding author on reasonable request.
